# Effect of change in individual and household level characteristics on anemia prevalence among adolescent boys and girls in India

**DOI:** 10.1186/s12889-022-13863-w

**Published:** 2022-08-03

**Authors:** Shobhit Srivastava, Pradeep Kumar, Ronak Paul, Paramita Debnath

**Affiliations:** 1 Department of Survey Research & Data Analytics, International Institute for Population Sciences, Mumbai, Maharashtra India; 2 Department of Public Health & Mortality Studies, International Institute for Population Sciences, Mumbai, Maharashtra 400088 India; 3 Department of Fertility Studies, International Institute for Population Sciences, Mumbai, Maharashtra India

**Keywords:** Anemia, Adolescent boys, Adolescent girls, Random-effect, UDAYA

## Abstract

**Background:**

Anemia is a significant public health challenge that affects the population of all nations. Anemia among adolescents emerged as an alarming public health issue as it harms an individual’s physical capacity and cognitive and work performance. The study aims to determine the effect of changes in individual and household level factors on the prevalence of anemia among adolescent boys and girls.

**Method:**

The study utilized data from two waves of the “Understanding the lives of adolescent and young adults” (UDAYA) survey, conducted in Bihar and Uttar Pradesh during 2015–16 (wave-1) and 2018–19 (wave-2). The sample size for the present study was 4216 and 5974 unmarried adolescent boys and girls aged 10–19 years in both waves. We performed descriptive analysis to observe the characteristics of adolescents during 2015–16. Further, changes in selected independent variables from wave-1 to wave-2 were examined using the proportion test. Moreover, random-effect regression models were employed to examine the association of changes in individual and household level factors with anemia prevalence among adolescents.

**Results:**

The prevalence of anemia decreased over time among adolescent boys (33 to 30%), whereas it increased among adolescent girls (59 to 63%). The results from the random-effect model show that adolescent boys who used shared toilets were more anemic than those who used a private restroom [β:0.05, 95% CI:(0.01, 0.08)]. Moreover, underweight [β:0.05, CI:(0.01, 0.09)] and thin [β:0.04, CI:(0.00, 0.07)] adolescent boys were more likely to be anemic compared to their normal counterparts. Additionally, boys who belonged to the poorest [β:0.08, CI:(0.02, 0.14)] households had a higher risk of anemia than the richest household.

**Conclusion:**

The anemia prevalence was higher among adolescents aged 10–19 years in Uttar Pradesh and Bihar. This study has filled an information gap by providing state-level representative estimates indicating underweight status and thinness as the common factors behind the anemia prevalence among adolescent boys than in girls. Iron deficiency anemia is the most prevalent in certain age groups in India. Hence, Anemia prevention efforts and iron-folic acid (IFA) supplementation programs are currently being strengthened in India, targeting the high-risk population.

**Supplementary Information:**

The online version contains supplementary material available at 10.1186/s12889-022-13863-w.

## Background

Anemia is a significant public health challenge that affects the population of all nations [1]. Across the globe, one-fourth of the world’s population suffers from anemia. One in four school-going children and four in every ten women are affected by it [1, 2]. Although the global burden of anemia has declined from 2007 to 2017, it still accounted for 34 million years lived with a disability in 2017 [3]. The World Health Organization (WHO) defines anemia as the condition where the percentage of red blood cells and consequently the oxygen-carrying capacity of the blood drops alarmingly and leads to a situation where the body’s physiological requirements are not fulfilled [4, 5]. Prolonged exposure to anemia leads to detrimental consequences like increased susceptibility to infections (due to immunity decline), maternal and child deaths, cognitive and physical impairment, and a decline in work productivity among adults [6–8]. Iron deficiency is the most common cause of anemia [1, 2, 5]. In contrast, the other causes of anemia include nutritional deficiencies (vitamin A, vitamin B12, copper and folic acid), parasitic infections, genetic disorders that affect hemoglobin synthesis, decreased red blood cell production, blood loss and chronic ailments [1, 2, 5]. Although half of all anemia cases can be attributed to iron deficiency, this percentage is more significant among adolescents [2, 9].

Adolescence is a phase in a person’s life characterized by different bodily changes. The WHO defines adolescents as people between 10–19 years of age who comprise 16% of the world’s population [10, 11]. While the proportion of adolescents is higher (20%) among countries in the South-East Asia Region, so is the prevalence of anemia in this region [12, 13]. Anemia prevalence is higher in India, where six out of ten adolescent girls are anemic [13]. According to National Family Health Survey 2015–16, India accounts for 29 and 54% of anemic boys and girls in 15–19 years, respectively [14].

Anemia among adolescents has emerged as an alarming public health issue as it harms an individual’s physical capacity, cognitive and work performance [13, 15]. One of India’s typical forms of anemia is iron deficiency anemia (IDA), prevalent among one in every five adolescents [16]. The risk of IDA is higher in both adolescent girls and boys in India [15]. Some Indian studies show that girls who experience heavy menstrual bleeding at the start of their menarche are more prone to develop anemia during adolescence [17–19]. This unfavorable situation can worsen further when the adolescent girls are socially entwined in early marriage and adolescent pregnancy. Subsequently, it increases the risk of child and maternal mortality, preterm labor, low birth weight and different health issues in adolescents [20].

Further, adolescent boys are also not spared from the consequences of iron deficiency anemia. As increment of body mass, muscle and expansion of blood volume increase their iron requirement in adolescence, lack of which can affect their growth and development [21, 22]. Two small-scale studies from India have also pointed to girls’ vulnerability from Scheduled Tribe social groups and those residing in rural communities towards becoming anemic [21, 23]. The same studies also provide evidence of the increasing prevalence of anemia with the increasing age among adolescent girls and decreasing with adolescent boys’ growing age, respectively. Therefore, multiple factors such as age, years of schooling, lower body weight, and other relatable factors such as people belonging to lower socioeconomic stratum, lower social standard, rural place of residence and unhygienic household environment lead to frequent parasite infestation, which further contributes to anemia and iron deficiency [24–28]. Studying the importance of each of these factors contributing to levels of anemia among adolescents is crucial for the development of essential strategies to reduce anemia prevalence in this age group [17, 18, 29]. Some studies also highlighted the role of community-level interventions in increasing awareness and reducing the prevention of IDA among adolescents [30, 31]. Furthermore, one study found anthropometric failure to be a significant predictor of anemia among adolescents in lower-middle-income countries [8]. Few studies have also shown that inadequate intake of iron-rich food and weekly supplementation of iron-folic acid tablets had shown a consistent increment of anemia among adolescents [13, 32–34].

The prevalence of anemia among pregnant women, adolescent boys and girls, and under-five children has always been India’s persistent public health challenge [35]. Therefore, the government has taken several initiatives such as the “Iron Plus initiative”, distribution of iron-folic acid tablets among pregnant women, “Poshan Abhiyaan” and “Anemia Mukt Bharat strategy” to bring down the national prevalence of anemia [36, 37]. As a result of these policy-level interventions, a new impetus is given to address anemia, but the efforts are partially successful [38]. Such slow progress is insufficient to make India anemia free by 2030 [36]. Anemia is still highly prevalent in the Indian states of Uttar Pradesh and Bihar [35]. Extant literature in the Indian context was limited to showing predictors of anemia among adolescent girls, which may potentially underestimate the effect of anemia on adolescent boys. This gives us the rationale for this study, which examines the factors associated with anemia among adolescent boys and girls. Further, panel data allows for examining anemia prevalence among adolescents in the high-risk states of Uttar Pradesh and Bihar over time. This study aims to determine how altering individual and family level variables affect the prevalence of anemia in adolescent boys and girls. The study examined the null hypothesis that there was no effect of changes in individual and household factors on the prevalence of anemia among adolescent boys and girls.

## Methods

### Data

The study utilized data from “Understanding the Lives of Adolescent and Young Adults” (hereafter UDAYA), the longitudinal study on adolescents aged 10–19 in Bihar and Uttar Pradesh [38]. The first wave was conducted in 2015–16, and the follow-up survey was conducted after three years in 2018–19. Unmarried boys and girls aged 10–19 years were interviewed, as were married girls aged 15–19 years. The study used a multi-stage stratified sampling technique to draw sample areas separately for rural and urban areas. In each state, 150 primary sampling units (PSUs)—villages in rural regions and census wards in urban areas—were chosen as the sample frame, based on the 2011 census list of villages and wards. In each PSU, interviewee households were selected by systematic sampling. More information about the study’s design and sampling technique may be found elsewhere [38].

In wave-1 (2015–16), 20,594 adolescents (adolescent girls: 14,160 and adolescent boys: 6,434) were interviewed using the structured questionnaire with a response rate of 92%. Moreover, in wave-2 (2018–19), the study again interviewed the participants who were successfully interviewed in 2015–16 and consented to be re-interviewed. Of the 20,594 eligible for the re-interview, the survey re-interviewed 4,567 unmarried boys and 12,251 girls (both married and unmarried). After excluding the respondents who gave an inconsistent response to age and education in the follow-up survey (3%), the final follow-up sample covered 4,428 boys and 11,864 girls, with a follow-up rate of 74% for boys and 81% for girls [38]. The sample size for the present study was 4216 and 5974 unmarried adolescent boys and girls aged 10–19 years in wave-1 and wave-2. We dropped the cases lost to follow-up from the sample to balance the dataset [39].

### Outcome variable

Three levels of severity of anemia were distinguished: mild anemia (10–11.4 g/dl for 10–11-year-olds, 10–11.9 g/dl for 12–14-year-olds and non-pregnant girls in ages 15–19 years, 10–10.9 g/dl for pregnant girls in ages 15–19 years, and 12.0–12.9 g/dl for boys in ages 15–19 years); moderate anemia (7.0–9.9 g/dl for 10–14-year-olds and girls in ages 15–19 years, regardless of pregnancy status at the time of the interview, and 9.0–11.9 g/dl for boys in ages 15–19 years); and severe anemia (< 7.0 g/dl for 10–14-year-olds and girls in ages 15–19, regardless of pregnancy status, and < 9.0 g/dl for boys in ages 15–19 years) [38]. The variable was coded as 0 “non-anemic” and 1 “anemic,” including mild/moderate/severe anemia. The analysis was further bifurcated into adolescent boys and girls as the data provide estimates separately for both categories [38].

### Explanatory variables

The explanatory variables were grouped into household environmental factors, individual factors, and household factors.

#### Household environment factors

1. The Source of drinking water was coded as “piped source” and “others” [40]. “Others” include open wells, surface water/river/stream/pond and tanker trucks.
2. The Source of cooking fuel was coded as “unclean” and “clean” [40]. Unclean includes Wood/crop residue/dung cakes/coal/charcoal, kerosene and Others. Clean fuel includes Electricity, Liquid Petroleum Gas (LPG) and Bio-gas.
3. The type of toilet facility was coded as “Own flush/pit,” “shared flush/toilet,” and “others” [40]. Others include own pit toilet, share pit toilet, no facility and others.

#### Individual factors

1. The age of the respondent was taken as a continuous variable (10–19 years as wave-1)
2. Years of schooling were taken as a continuous variable.
3. Underweight was coded as “Yes” ((body mass index) BMI less than 18.5) and “No” (BMI 18.5 or more) [39].
4. Thinness was coded as “Yes” (BMI-for-age Z-score < -2SD)” and “No” (BMI-for-age Z-score ≥ -2SD) [39].
5. Received Iron folic acid (IFA) and deworming tablets were coded in no and yes.

#### Household factors

1. The wealth index was recoded as poorest, poorer, middle, richer and richest [39, 41].
2. Caste was recoded as Scheduled caste and Scheduled tribe (SC/ST), and non-SC/ST [42].
3. Religion was categorized as Hindu and non-Hindu. The category of non-Hindu was recoded to include all religions except Hindus as the frequency of other religions was very low [39].
4. The place of residence was available in data as urban and rural.
5. Data were available for two states, i.e., Uttar Pradesh and Bihar, as the survey was conducted in these two states only.

### Statistical analysis

Descriptive analysis was done to observe the characteristics of married adolescent girls at wave-1 (2015–16). Additionally, changes in certain selected variables were observed from wave-1 (2015–16) to wave-2 (2018–19), and the statistical significance was tested using the proportion test [43]. Moreover, random-effect regression analysis was used to estimate the association of change in prevalence of anemia with the changes in the household environment and individual factors [44, 45]. The estimates were presented as coefficients with a 95% confidence interval (CI). Throughout the manuscript, statistical significance was determined at the 5% level. This study applied the Hausman test to obtain a better model (fixed-effects or random-effect) for the analysis. Hausman test results confirmed that the random-effects model was more appropriate than the fixed-effects model for our analysis (Hausman test statistics were insignificant) [46, 47]. Detailed results of the Hausman test can be found in supplementary tables S1 and S2.

Additionally, the random-effect model has a particular benefit over the fixed-effect model for the present paper’s analysis. That advantage is its ability to estimate the effect of any variable that does not vary within an individual over time. This holds for all level 2 variables (e.g., wealth status is assumed constant for wave-1 and wave-2) [48–50]. Descriptive and longitudinal analysis was performed in STATA 14 software [51].

## Results

**Fig. 1:**
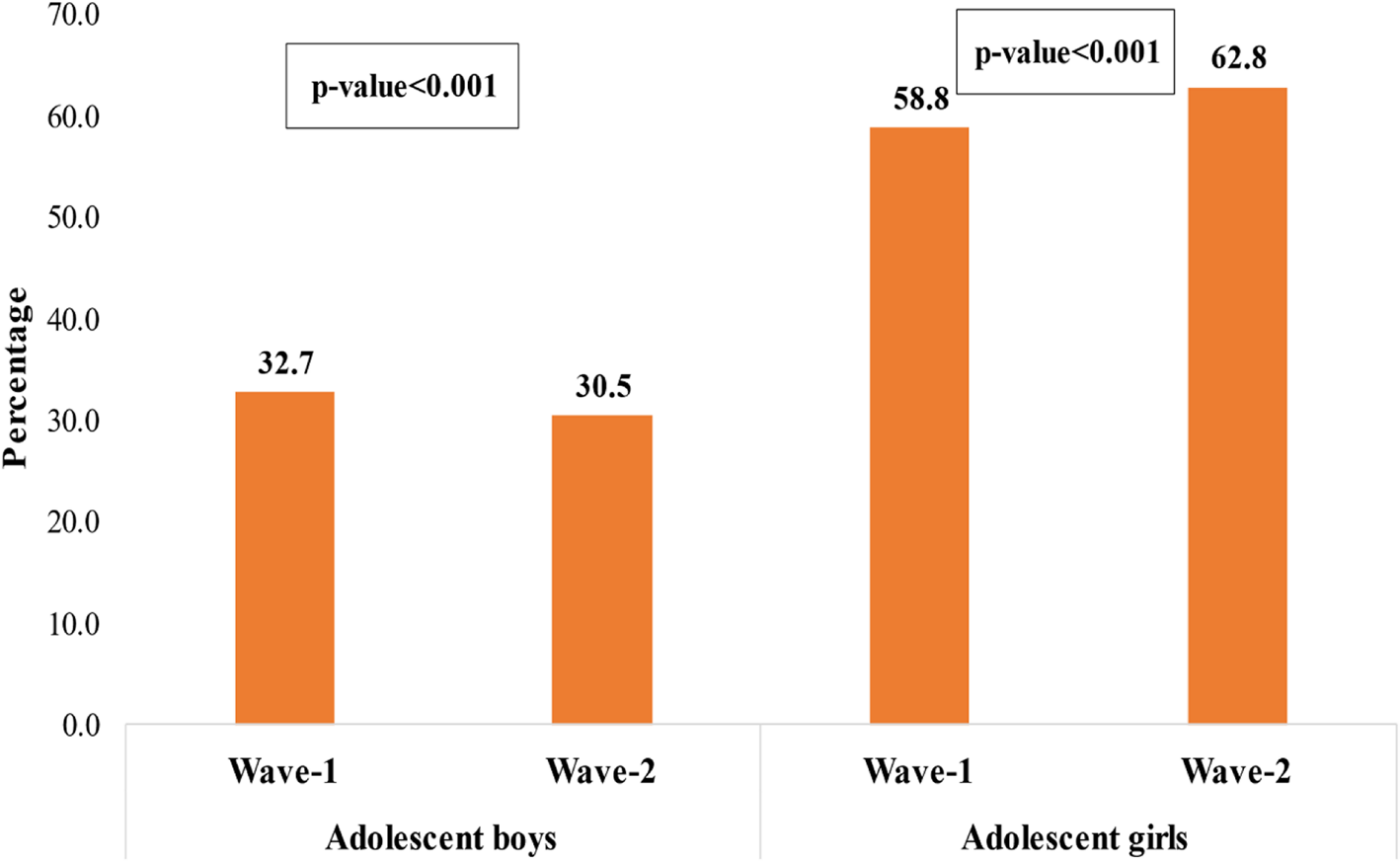
Prevalence of anemia among adolescent boys and girls. Wave-1:2015–16; Wave-2: 2018–19

Figure 1 shows that the prevalence of anemia declined significantly over time among boys (32.7 to 30.5%; *p* < 0.001), whereas it increased significantly among adolescent girls over time (58.8 to 62.8%; *p* < 0.001). Table 1 shows a higher proportion of adolescents were Hindu (boys-84.8% and girls-78%), and about one-fourth of adolescents (26% boys and 24% girls) belonged to scheduled caste/scheduled tribe (SC/ST). Most adolescents lived in rural areas (boys-85.1% and girls-78.4%). Figure-S1 reveals the prevalence of anemia among adolescent boys and girls by severity level (see supplementary file).

**Table 1:** Socioeconomic characteristics of the study population, 2015–16

Background characteristics	Adolescent boys		Adolescent girls	
	**Sample**	**Percentage**	**Sample**	**Percentage**
**Wealth index**				
Poorest	548	13.0	838	14.0
Poorer	861	20.4	1052	17.6
Middle	947	22.5	1254	21.0
Richer	958	22.7	1494	25.0
Richest	902	21.4	1335	22.4
**Religion**				
Hindu	3576	84.8	4662	78.0
Non-Hindu	640	15.2	1312	22.0
**Caste**				
SC/ST	1105	26.2	1407	23.6
Non-SC/ST	3111	73.8	4567	76.4
**Place of residence**				
Urban	627	14.9	1293	21.6
Rural	3589	85.1	4681	78.4
**States**				
Uttar Pradesh	2751	65.3	3393	56.8
Bihar	1465	34.8	2581	43.2
**Total**	4216	100.0	5974	100.0

*SC/ST* Scheduled Caste/Scheduled Tribe

In Table 2, mean age of adolescents in wave-1 was 13–14 years, and in wave-2, it was 16–17 years. Similarly, adolescents’ mean years of schooling were six and eight years in wave-1 and wave-2, respectively. Moreover, the percentage of underweight adolescents (BMI < 18.5) decreased in the last three years (boys: 86.2 to 66.8%, and girls: 83.9 to 58.1%). Similarly, the prevalence of thinness among adolescents also declined (boys-28.4 to 22.5% and girls-19.2 to 12.2%). Moreover, the consumption of IFA tablets increased over the period from wave-1 to wave-2 (boys-23.8 to 27.5% and girls-25.8 to 32.2%).

**Table 2:** Summary statistics of explanatory variables used in the analysis of UDAYA wave-1 and wave-2

**Variables**	**Adolescent boys**			**Adolescent girls**		
	**wave-1**	**wave-2**	***p*-value**	**wave-1**	**wave-2**	***p*-value**
Piped water source	96.0	92.5	< 0.001	97.9	95.7	< 0.001
Clean cooking fuel	20.5	32.0	< 0.001	22.1	33.2	< 0.001
Own flush/pit toilet	31.5	57.4	< 0.001	35.3	56.7	< 0.001
Mean age (years)	13.9	16.8	0.789	13.3	16.2	< 0.001
Mean schooling (years)	6.7	8.8	0.090	6.2	8.3	< 0.001
Underweight (BMI > 18.5)	86.2	66.8	< 0.001	83.9	58.1	< 0.001
Thinness	28.4	22.5	0.015	19.2	12.2	< 0.001
Consumption of IFA tablets	23.8	27.5	0.173	25.8	32.2	< 0.001
N	4216	4216		5974	5974	

*BMI* Body mass index, *IFA* Iron folic acid, *wave-1* 2015–16, *wave-2* 2018–19

*p*-values are based on t-test and proportion test

The prevalence of anemia among adolescent boys and girls by their background characteristics is presented in Table 3. The prevalence of anemia increased by 11% among adolescent boys who suffered from thinness (33.5 to 44.4%). Moreover, anemia prevalence increased by 11% among those who belonged to the middle wealth quintile (29.3 to 40%), non-Hindu (25.3 to 30.6%), who lived in Bihar state (28.4 to 31.4%), and those families used unclean cooking fuel (33.4 to 35.3%). Moreover, anemia prevalence was higher among adolescent girls who used other sources of drinking water (40.4 to 69.6%), did not consume IFA tablets (54.6 to 66.3%), belonged to rural areas (57.6 to 64.4%), non-Hindu (53.1 to 65.1%), and lived in Bihar (57.8 to 72%). The prevalence of anemia has decreased by 2.8% among boys (35.4 to 32.6%) and increased by 5% among girls (55.1 to 60.1%) in Uttar Pradesh. Moreover, Bihar saw a 3% decline in anemia prevalence among boys (28.4 to 31.4%), and it increased by 14% among girls (57.8 to 72%).

**Table 3:** Percentage of anemia among adolescent boys and girls by their background characteristics

Variables	Adolescent boys			Adolescent girls		
	**Wave-1**	**Wave-2**	***p*-value**	**Wave-1**	**Wave-2**	***p*-value**
**Household environment**						
**Source of drinking water**						
Others	42.7	28.5	< 0.001	40.4	69.6	< 0.001
Piped	32.3	32.4	< 0.001	56.7	65.5	< 0.001
**Source of cooking fuel**						
Unclean	33.4	35.3	< 0.001	57.3	64.9	< 0.001
Clean	30.1	24.8	< 0.001	52.3	67.4	< 0.001
**Type of toilet facility**						
Own flush/pit	28.0	29.6	< 0.001	55.5	66.1	< 0.001
Shared flush/toilet	32.2	29.9	< 0.001	52.0	64.8	< 0.001
Others	34.9	36.2	< 0.001	57.2	65.2	< 0.001
**Individual characteristics**						
**Underweight**						
No	26.2	24.3	< 0.001	56.6	66.3	< 0.001
Yes	33.7	36.4	< 0.001	56.3	65.2	< 0.001
**Thinness**						
No	32.4	30.2	< 0.001	57.6	67.1	< 0.001
Yes	33.5	44.4	< 0.001	51.1	56.5	< 0.001
**Consumption of IFA tablets**						
No	33.4	31.4	< 0.001	54.6	66.3	< 0.001
Yes	30.9	33.7	< 0.001	60.5	64.4	< 0.001
**Household characteristics**						
**Wealth index**						
Poorest	42.5	36.8	< 0.001	59.8	65.7	< 0.001
Poorer	31.7	35.0	< 0.001	55.5	63.8	< 0.001
Middle	29.3	40.0	< 0.001	58.5	73.6	< 0.001
Richer	35.0	26.1	< 0.001	49.9	62.1	< 0.001
Richest	28.6	22.9	< 0.001	60.0	63.1	< 0.001
**Religion**						
Hindu	34.0	32.5	< 0.001	57.2	65.8	< 0.001
Non-Hindu	25.3	30.6	< 0.001	53.1	65.1	< 0.001
**Caste**						
SC/ST	36.0	35.4	< 0.001	57.6	64.4	< 0.001
Non-SC/ST	31.6	31.0	< 0.001	55.9	65.6	< 0.001
**Place of residence**						
Urban	26.2	22.2	< 0.001	57.6	64.9	< 0.001
Rural	33.8	33.7	< 0.001	56.1	65.8	< 0.001
**States**						
Uttar Pradesh	35.4	32.6	< 0.001	55.1	60.1	< 0.001
Bihar	28.4	31.4	< 0.001	57.8	72.0	< 0.001

Estimates for age and schooling were not presented as they were continuous

*p*-values are based on the proportion test

Table 4 shows the estimated effects of explanatory variables on anemia from fixed and random-effect models. The random-effects model shows that household environment factors had no effects on anemia among adolescents except for types of toilet facilities. For instance, adolescent boys who used shared flush/toilets were more anemic compared to those who used their own flush/pit toilets (β = 0.05, *p* < 0.10). The age of adolescent boys was not associated with anemia. However, with an increase in age, anemia was increased by 0.02 units among adolescent girls (*p* < 0.10). Moreover, with the increasing level of education, the anemia prevalence decreased by 0.02 units among adolescent boys (*p* < 0.10). Underweight adolescent boys had a higher risk of anemia than those who were not underweight (β = 0.05, *p* < 0.10).

**Table 4:** Estimated effects of explanatory variables on the anemia from fixed and random-effect models

Variables	Adolescent boys (10–19)		Adolescent girls (10–19)	
	**Fixed-effect**	**Random-effect**	**Fixed-effect**	**Random-effect**
**Household environment**				
**Source of drinking water**				
Others	0.04(-0.08,0.15)	0.01(-0.07,0.09)	0.01(-0.13,0.16)	-0.04(-0.15,0.06)
Piped	Ref	Ref	Ref	Ref
**Source of cooking fuel**				
Unclean	-0.01(-0.07,0.05)	-0.01(-0.05,0.03)	-0.01(-0.08,0.07)	0.02(-0.03,0.07)
Clean	Ref	Ref	Ref	Ref
**Type of toilet facility**				
Own flush/pit	Ref	Ref	Ref	Ref
Shared flush/toilet	0.11*(-0.19,-0.02)	0.05*(0.01,0.08)	0.02(-0.07,0.11)	0.02(-0.04,0.08)
Others	0.01(-0.04,0.07)	0.02(-0.02,0.06)	0.01(-0.06,0.08)	0.01(-0.04,0.05)
**Individual characteristics**				
**Age**	0.06(0,0.13)	0.01(-0.01,0.01)	-0.03(-0.12,0.05)	0.02*(0.01,0.03)
**Schooling**	-0.01(-0.04,0.01)	-0.02*(-0.03,-0.01)	0.01(-0.03,0.03)	0.01(-0.01,0)
**Underweight**				
No	Ref	Ref	Ref	Ref
Yes	0.05(-0.01,0.11)	0.05*(0.01,0.09)	0.02(-0.05,0.08)	0.01(-0.03,0.05)
**Thinness**				
No	Ref	Ref	Ref	Ref
Yes	0.02(-0.04,0.09)	0.04*(0,0.07)	-0.06(-0.14,0.03)	-0.02(-0.07,0.03)
**Consumption of IFA tablets**				
No	Ref	Ref	Ref	Ref
Yes	-0.01(-0.06,0.04)	0.01(-0.04,0.03)	0.01(-0.05,0.06)	0.01(-0.02,0.05)
**Household characteristics**				
**Wealth index**				
Poorest		0.08*(0.02,0.14)		-0.02(-0.09,0.06)
Poorer		0.03(-0.03,0.08)		0.01(-0.07,0.07)
Middle		0.05*(-0.01,0.1)		-0.03(-0.09,0.04)
Richer		0.04*(-0.01,0.08)		-0.02(-0.07,0.04)
Richest		Ref		Ref
**Religion**				
Hindu		Ref		Ref
Non-Hindu		-0.04(-0.08,0)		-0.01(-0.05,0.04)
**Caste**				
SC/ST		Ref		Ref
Non-SC/ST		-0.02(-0.06,0.01)		-0.01(-0.05,0.03)
**Place of residence**				
Urban		Ref		Ref
Rural		0.05*(0.02,0.09)		-0.02(-0.06,0.03)
**States**				
Uttar Pradesh		Ref		Ref
Bihar		-0.06*(-0.09,-0.03)		0.05*(0.01,0.08)
**Year**				
2015–16	Ref	Ref	Ref	Ref
2018–19	-0.15(-0.35,0.05)	0.04*(0.01,0.07)	0.18(-0.07,0.42)	0.05*(0.01,0.08)
Sigma_u	0.408	0.167	0.425	0.229
Sigma_e	0.404	0.405	0.429	0.430
rho	0.505	0.146	0.495	0.220
F-stat	1.68		2.79**	
Wald chi-square	163.54***			48.3***
Haussmann test	11.72		6.67	

^*^if *p* < 0.10, **if *p* < 0.05, ***if *p* < 0.001

*Ref* Reference, *BMI* Body mass index, *SC/ST* Scheduled Caste/Scheduled Tribe, *IFA* Iron folic acid, *wave-1* 2015–16

In contrast, boys who suffered from thinness were more likely to be anemic than those who did not suffer (β = 0.04, *p* < 0.10). Compared to the richest household, the risk of anemia was higher among boys who belonged to the poorest (β = 0.08, *p* < 0.10), middle (β = 0.05, *p* < 0.10), and richer (β = 0.04, *p* < 0.10) household. Similarly, adolescent boys in rural areas had a significantly higher risk of anemia than those in urban areas (β = 0.05, *p* < 0.10).

## Discussion

The study used longitudinal data and robust statistical methods (random-effect and fixed-effect model) to estimate the consequences of changes in the household environment and individual factors on differences in the prevalence of anemia among 10–19 years of adolescent boys and girls. However, the present study focused on the often-overlooked population group in India at risk of anemia, namely adolescent boys and girls [52]. The present study found that the prevalence of anemia was 30.5% and 62.8% among adolescent boys and girls, respectively. The prevalence rate of anemia was more pronounced among girls than boys and witnessed a rise in wave-2 for girls. According to the WHO’s classification of anemia as a problem of public health significance, the prevalence of anemia in our study population would be classified as (20.0–39.9%) moderate public health concern for boys and (> 40.0%) severe public health concern for girls [53]. Nevertheless, extant studies predominantly suggested anemia to be expected in children, adolescent girls and boys, and young pregnant women, considering them a high-risk group in developing countries [54]. Moreover, we found that increasing age was statistically associated with an increased likelihood of anemia, especially among girls than in boys. It indirectly indicates the occurrence of menarche, followed by high menstrual losses in later stages of puberty, increasing the risk of anemia.

In developing countries like India, anemia is primarily due to nutritional problems in the adolescent age [55]. The study found that there are more underweight adolescent boys (66.8%) compared to girls (56.8%), and the prevalence of thinness was also higher in boys (22.5%) than in girls (12.2%). These estimates indicate a dramatically different level of nutritional status for adolescents in Bihar and Uttar Pradesh. Additionally, underweight and thin adolescent boys were highly susceptible to anemia. Thus, the nutritional status of boys inflates the overall prevalence of anemia in boys.

In contrast, the prevalence of anemia remains unchanged with girls’ increasing underweight status and thinness. Earlier studies on other Asian countries with comparable nutritional indicators suggested similar findings [56]. Also, improperly balanced diet intake and nutritional assessment before consumption lead to iron deficiency. The iron requirement is accelerated for growth needs and development [57]. Lack of iron often leads to severe anemia in this age group and has been an indicator of long-term adverse impact on overall health due to increased vulnerability to infections and weak immunity [30]. To control and prevent the prevalence of anemia, the government of India launched a weekly iron-folic acid (IFA) supplementation program (WIFS), which instructed adolescents to consume iron folic supplements once a week [58]. Interestingly, the consumption of IFA tablets had no significant difference in the prevalence of anemia in adolescent boys and girls.

In line with a few studies, this longitudinal investigation showed that education strongly correlates with anemia as adolescents with a higher level of education are more open to new information on personal hygiene and healthy nutritional practices [59]. We observed a decrease in the prevalence of anemia among adolescent boys with an increase in their education level. In contrast, this association remained unaffected amongst the adolescent girls, although the mean years of schooling are almost similar for both sexes (8.8 and 8.3 years for boys and girls, respectively). In the present study, anemia prevalence is unevenly distributed in all socioeconomic groups. It is found to be highest among adolescents in the poorest wealth quintile, which is in line with most of the past studies as the risk of anemia among them depends on various factors such as availability and affordability of food high in iron, folic and vitamins, which highly contributes to the problem [59]. Also, boys who used shared toilet facilities were at higher risk of anemia. Higher socioeconomic status and wealth quintile were perceived as protective effects of anemia. The finding of our study was not in concordance with previous studies, as middle and richer wealth quintiles were also at high risk of anemia [56]. It explains that unhealthy nutritional practices (junk food consumption) among adolescents in the higher wealth quintile might also increase the prevalence of anemia. Overall, the severity of anemia was most elevated among rural male adolescents compared to that of urban adolescents. To end with, the study results demonstrated that the prevalence of anemia is very high among adolescents, especially in Bihar, where girls have a higher prevalence of anemia than boys. This study indicated the importance of adolescence as a phase to reduce the risk of anemia and overall health through appropriate interventions in this critical age group.

The study has strengths and limitations. The study used longitudinal data to observe the change in the anemia prevalence among the same population. Moreover, the study is based on the two largest states of India: Uttar Pradesh and Bihar, which has a home of every fourth adolescent in India. On the other hand, the study did not check interaction effects, and future studies can do the same. In addition, the predictors used in this study for two high prevalent Indian states with low mean age at marriage. Therefore, results might differ for other states.

## Conclusion

We found that anemia was a severe public health problem among adolescents aged 10–19 years in Uttar Pradesh and Bihar. This study has filled an information gap by providing state-level representative estimates indicating underweight status and thinness as the most common causes that contributed to the prevalence of anemia among adolescent boys than in girls. Iron deficiency anemia is the most prevalent in certain age groups in India. Hence, anemia prevention efforts and IFA supplementation programs are being strengthened in India, targeting the high-risk population. However, our study shows no effectivity of IFA tablet consumption in reducing the risk of anemia in this age group. Integrating interventions that mainly focus on this high-risk adolescent population is significant for reducing micronutrient deficiency and improving overall health in the later critical ages.

## Supplementary Information

**Additional file 1:** **Figure-S1.** Prevalence of anaemia among adolescent boys and girls by severity level. **Table-S1.** Hausman test results for adolescent boys. **Table-S2.** Hausman test results for adolescent girls.

## Data Availability

Data was collected as part of the Population Council’s UDAYA study, which is publicly available on the site of Harvard Dataverse at: https://dataverse.harvard.edu/dataset.xhtml?persistentId=doi:10.7910/DVN/RRXQNT

## Abbreviations

OR: Odds Ratio
CI: Confidence Interval
UDAYA: Understanding the Lives of Adolescents and Young Adults
SC: Scheduled Caste
ST: Scheduled Tribe
