# Supplementary material for: Effect of change in individual and household level characteristics on anemia prevalence among adolescent boys and girls in India
Source: BMC Public Health. 2022 Aug 3;22:1478. doi: 10.1186/s12889-022-13863-w (PMC9351076; doi:10.1186/s12889-022-13863-w)
Supplement: Supplementary file 1 — Additional file 1: Figure-S1. Prevalence of anaemia among adolescent boys and girls by severity level. Table-S1. Hausman test results for adolescent boys. Table-S2. Hausman test results for adolescent girls. [file 12889_2022_13863_MOESM1_ESM.docx]

**Figure-S1. Prevalence of anaemia among adolescent boys and girls by severity level. Wave-1:2015-16; wave-2: 2018-19.**

| **Table-S1.** Hausman test results for adolescent boys | | | | |
| --- | --- | --- | --- | --- |
| **Variables** | **(b)** | **(B)** | **(b-B)** | **sqrt(diag(V_b-V_B))** |
|  | **fe** | **re** | **Difference** | **S.E.** |
| Piped water source | 0.038 | 0.009 | 0.029 | 0.042 |
| Clean cooking fuel | -0.010 | -0.011 | 0.001 | 0.026 |
| Own flush/pit toilet |  |  |  |  |
| Shared flush/toilet | -0.106 | -0.018 | -0.088 | 0.032 |
| Others | 0.012 | 0.019 | -0.007 | 0.021 |
| Age | 0.062 | 0.005 | 0.057 | 0.034 |
| Schooling | -0.014 | -0.020 | 0.006 | 0.012 |
| Underweight | 0.052 | 0.051 | 0.001 | 0.025 |
| Thinness | 0.024 | 0.039 | -0.014 | 0.029 |
| Consumption of IFA | -0.012 | -0.005 | -0.007 | 0.018 |
| Year | -0.149 | 0.037 | -0.186 | 0.101 |

**Note –** fe: Fixed effects, re: Random effects.

| **Table-S2.** Hausman test results for adolescent girls | | | | |
| --- | --- | --- | --- | --- |
| **Variables** | **(b)** | **(B)** | **(b-B)** | **sqrt(diag(V_b-V_B))** |
|  | **fe** | **re** | **Difference** | **S.E.** |
| Piped water source | 0.013 | -0.043 | 0.056 | 0.050 |
| Clean cooking fuel | -0.008 | 0.019 | -0.027 | 0.030 |
| Own flush/pit toilet |  |  |  |  |
| Shared flush/toilet | 0.022 | 0.020 | 0.002 | 0.035 |
| Others | 0.008 | 0.008 | 0.000 | 0.027 |
| Age | -0.035 | 0.017 | -0.052 | 0.043 |
| Schooling | 0.001 | -0.004 | 0.005 | 0.014 |
| Underweight | 0.016 | 0.009 | 0.007 | 0.025 |
| Thinness | -0.057 | -0.020 | -0.037 | 0.034 |
| Consumption of IFA | 0.003 | 0.015 | -0.012 | 0.021 |
| Year | 0.177 | 0.046 | 0.131 | 0.124 |

**Note –** fe: Fixed effects, re: Random effects.
